# Book Review: New World Monkeys – The Evolutionary Odyssey

**DOI:** 10.5194/pb-8-15-2021

**Published:** 2021-05-21

**Authors:** EckhardÂ W. Heymann

**Affiliations:** Verhaltensökologie & Soziobiologie, Deutsches Primatenzentrum, Leibniz-Institut für Primatenforschung, Göttingen, Germany

*Rosenberger, A. L.: New World Monkeys – The Evolutionary Odyssey, Princeton University Press, Princeton, USA, xviii + 334 pp., ISBN 978-0-691-14364-4, USD 39.95 (hardcover), 2020.*

I like this book, not because I would agree with everything that is written in it, but because it provides an excellent overview of the biology of our more distant cousins, the New World monkeys (hereafter NWM), because it enriches highly digestible scientific information with accounts of personal experiences, and last but not least because it is a stimulus for controversial discussion, particularly on the evolutionary origin and taxonomy of NWM.

The preface consists of a short autobiographical journey through the history of research on NWM. Here and in other places in the book, Rosenberger highlights how important experiences during field studies under the guidance of the late Warren G. Kinzey, one of the pioneers of NWM field research, were for his own career and scientific thinking. At this point, one wishes that more students and researchers could have such experiences (before it is too late) even though they are not determined to become field researchers but to base their work in labs and museums. The following 11 chapters provide a broad panorama of NWM, starting with the question (Chapter 1) “What is a New World monkey?” through chapters that discuss issues of taxonomy, evolutionary models, ecology, functional morphology, and social organisation, and the question of “How did platyrrhine ancestors get there [South America]?” (Chapter 10) and ending with a gloomy look at the many threats, particularly the recent increase in forest destruction, that endanger the future of NWM.

Extant adaptations cannot be understood without taking phylogenetic history and evolutionary contingencies into account. This consideration lies at the heart of the “Ecophylogenetic Hypothesis” to which Rosenberger adheres. Ecophylogenetics is the integration of “evolution and historical contingencies into the ecological research agenda through the widespread use of phylogenetic data” (Mouquet et al., 2012, p. 769). To this, Rosenberger adds the “Long-lineage Hypothesis” which posits that many “kinds of monkeys we see today … have existed for at least 20 million years with little change in their ecological situation” (p. 2). That the different families of NWM represent long lineages is supported by both morphological and genetic data. Whether “their ecological situation” has changed only very little over such a long period is perhaps more daring to state. A look into chapters of “Amazonia: landscape and species evolution – a look into the past” (Hoorn and Wesselingh, 2010) reveals quite some dynamics in ecological conditions in Amazonia, the region which harbours the highest diversity of NWM. However, does the ecological flexibility that we see in many extant NWM, e.g. the presence of howler monkeys in very humid lowland rainforests of Amazonia, very dry forests in eastern Brazil, and relatively open “llanos” forests in Venezuela not suggest that little ecological change is not a necessary condition for maintaining fundamental adaptations? Anyway, the ecological diversity of NWM, both extinct and extant, is nicely illustrated in this book.

Earlier on, Rosenberger had already expressed his disagreement with new taxonomic arrangements of NWM (Rosenberger, 2012). In his book he continues to argue, specifically against the splitting of genera and implicitly against the upgrading of subspecies to species. However, in several places, I found his lines of reasoning inconsistent if not contradictory. On the one hand, Rosenberger argues against the recently made split of the genus *Callicebus* into *Callicebus*, *Cheracebus* and *Plecturocebus* (Boubli et al., 2019) because of the lack of “evidence that the groups identified are in any way distinguished in lifestyle” (p. 63). I admit that we still know very little about the ecology and behaviour of titi monkeys and whether and how strong lineages and species differ; I could not currently name any significant ecological and behavioural differences. However, on the other hand, despite strong differences in lifestyle between large-bodied tamarins (e.g. *Saguinus mystax* and *Saguinus labiatus*) and the small-bodied tamarins (genus *Leontocebus*), summarised in Rylands et al. (2016), Rosenberger insists on retaining the small-bodied tamarins in the genus *Saguinus*, even though he states that *Saguinus nigricollis* (*Leontocebus nigricollis*) has a foraging style more similar to lion marmosets (genus *Leontopithecus*), associated with hands and fingers that are relatively longer than in other tamarins; these characteristics obviously set *L. nigricollis* (and the ecologically highly similar if not identical congeneric species) apart from *Saguinus*. Actually, differences in lifestyle between *Saguinus* and *Leontocebus*, e.g. differential use of forest strata and different prey-foraging technics (e.g. Nadjafzadeh and Heymann, 2008; Norconk, 1990), represent dimensions of the niches critical for allowing for their sympatry and formation of mixed-species associations throughout large parts of western Amazonia (Heymann and Buchanan-Smith, 2000). These differences between *Saguinus* and *Leontocebus* are fundamental, not only seasonal, as Rosenberger argues in contra of another taxonomic split, namely that of the genus *Cebus* into *Cebus* and *Sapajus*. In the end, all taxonomies are hypotheses, and only with the accumulation of evidence from all disciplines (morphology, genetics, ecology, etc.) can these hypotheses either become more solid or be replaced by more comprehensive hypotheses. Taxonomic controversies such as those sparked by Rosenberger are clearly helpful in the discussion.

The same is true for a second point of contention on taxonomic and phylogenetic issues, namely the position of night monkeys, *Aotus*, within the NWM tree. Rosenberger advocates a close relationship between Aotus and titi monkeys, placing them in the subfamily Homunculinae of the family Pitheciidae, along with *Homunculus*, a fossil primate from Miocene deposits in southern Argentina, arguing with supposed similarities, not only in morphology, but also in lifestyle. He proclaims them as an “outstanding example of a primate clade that is in some ways better defined by behaviour than by morphology” and emphasises pair living in particular as one of the shared “behavioral novelties” (p. 59) of *Aotus* and titi monkeys. However, in contrast to the claim that pair living is “in any case exceptionally rare among primates” (p. 270), this form of social organisation is more widespread amongst primates than in most other mammalian orders and probably was the ancestral state of the Anthropoidea (Kappeler and Pozzi, 2019; Lukas and Clutton-Brock, 2013).

Rosenberger's insistence on *Aotus* and titi monkeys being sister lineages is at odds with the genetic evidence which leaves titi monkeys with the Pitheciidae but affiliates *Aotus* with the cebids and callitrichids (e.g. Schneider and Sampaio, 2015). However, even phylogenies that combine morphological and genetic evidence separate *Aotus* and titi monkeys (Horovitz, 1999). Is the statement quoted above (“better defined by behaviour than by morphology”) the unintended concession that there is little morphological evidence for a close phylogenetic relationship between *Aotus* and titi monkeys?

By the time NWM evolved from a common ancestor with catarrhine primates, South America was an island. This has made the arrival of NWM ancestors an enigma (Ciochon and Chiarelli, 1980). Rafting on floating islands has been hypothesised as a mode of dispersal from Africa (e.g. Houlé, 1999). In Chapter 10, Rosenberger challenges this “Transatlantic Scenario” by estimating the probability of the different steps involved in such a voyage and concludes that it was extremely unlikely. As an alternative hypothesis, he postulates the “Americas Scenario” which involves “overland travel”, from Africa through Europe and through the “Thulean route” to North America, with an arrival time in South America at around 45 mya. However, does this scenario not also require crossing open seas – namely the Tethys between Africa and Europe and the Caribbean between North and South America? Distances to cross were probably shorter than the one between Africa and South America, but there were at least two seas to cross instead of only one in the transatlantic scenario. The “Thulean route” would have involved travel through subarctic or even arctic regions. While a warmer climate during that geological period would have potentially allowed primates to exist there, the 2-month periods where the sun does not rise over the horizon makes life for diurnal primates somewhat complicated. And finally, by the time ancestral NWM would have traversed North America, they would have had to co-exist with the rich communities of adapiforms and other primates. However, at least the “Americas Scenario” is a potentially testable hypothesis, since ancestral NWM should have left some fossils along their route, at least through North America. Conditions for fossilisation were apparently highly favourable in several regions of North America to even allow for the reconstruction of gradual changes in primate lineage (O'Leary, 2021). So, if NWM ancestors had made it through North America, fossils should show up at some moment.

Rosenberger's book is written in a highly accessible style which makes it readable also for a non-scientific audience, particularly because concepts, (taxonomic) principles and terminology are comprehensively explained in various places.

In conclusion, despite my criticism above, I highly recommend this book to everybody interested in New World monkeys, be it scientists, students in all stages of their scientific training or informed laymen. I am convinced that the controversial issues raised in this book will stimulate further research, bringing the “evolutionary odyssey” closer to a safe harbour of knowledge.
